# 5-hydroxymethylcytosine represses the activity of enhancers in embryonic stem cells: a new epigenetic signature for gene regulation

**DOI:** 10.1186/1471-2164-15-670

**Published:** 2014-08-09

**Authors:** Inchan Choi, Rinho Kim, Hee-Woong Lim, Klaus H Kaestner, Kyoung-Jae Won

**Affiliations:** Department of Genetics, Institute for Diabetes, Obesity and Metabolism, University of Pennsylvania, 3400 Civic Center Blvd, 19104 Philadelphia, PA USA

**Keywords:** 5hmC, GROseq, PolII, eRNA, mESC, Enhancer

## Abstract

**Background:**

Recent mapping of 5-hydroxymethylcytosine (5hmC) provides a genome-wide view of the distribution of this important chromatin mark. However, the role of 5hmC in specific regulatory regions is not clear, especially at enhancers.

**Results:**

We found a group of distal transcription factor binding sites highly enriched for 5-hdroxymethylcytosine (5hmC), but lacking any known activating histone marks and being depleted for nascent transcripts, suggesting a repressive role for 5hmC in mouse embryonic stem cells (mESCs). 5-formylcytosine (5fC), which is known to mark poised enhancers where H3K4me1 is enriched, is also observed at these sites. Furthermore, the 5hmC levels were inversely correlated with RNA polymerase II (PolII) occupancy in mESCs as well as in fully differentiated adipocytes. Interestingly, activating H3K4me1/2 histone marks were enriched at these sites when the associated genes become activated following lineage specification. These putative enhancers were shown to be functional in embryonic stem cells when unmethylated. Together, these data suggest that 5hmC suppresses the activity of this group of enhancers, which we termed “silenced enhancers”.

**Conclusions:**

Our findings indicate that 5hmC has a repressive role at specific proximal and distal regulatory regions in mESCs, and suggest that 5hmC is a new epigenetic mark for silenced enhancers.

**Electronic supplementary material:**

The online version of this article (doi:10.1186/1471-2164-15-670) contains supplementary material, which is available to authorized users.

## Background

5-hydroxymethylcytosine (5hmC) is an epigenetic mark that arises from oxidation of 5-methylcytosine (5mC) by Ten-eleven translocation (Tet) enzymes [1, 2]. The 5hmC mark has been studied in several cell types, such as mouse embryonic stem cells (mESCs) [2, 3], neuronal cells [4–6] and adipocytes [7]. 5hmC is enriched at promoters marked bivalently by H3K4me3 and H3K27me3 in mESCs [8], but depleted at promoters in the brain [9]. 5hmC is also enriched at specific transcription factor binding sites (TFBSs) in human and mouse ESCs [1, 9–14]. Specifically, in mESCs, 5hmC is depleted at Sox2 and Oct4 binding sites, but enriched for Esrrb and Tcfcp2l1 occupancy [12]. In human embryonic stem cells (hESCs), 5hmC is highly enriched at CTCF, Nanog, and Oct4 binding sites [11]. Another study in hESC observed that the 5hmC profile showed a bimodal distribution at Oct4, Sox2, TAF1 and p300 binding sites [9]. While these studies suggest a possible regulatory role for 5hmC at promoters and TFBSs, its function at these regulatory regions remains unclear.

Here, we report on a new repressive role for 5hmC at specific regulatory regions in mESCs. We show that 5hmC negatively correlates with nascent transcripts, especially at TFBSs. Interestingly, we discovered that a group of distal TFBSs displays a new epigenetic signature; these sites are exclusively enriched for 5hmC, depleted for activating histone modification marks (H3K4me1 and H3K27ac), and significantly reduced for nascent transcripts or enhancer RNAs (eRNAs). The expression of the genes close to these TFBSs was significantly lower than that of genes close to other classes of TFBSs. In addition, we found that a fraction of these TFBSs becomes enriched for activating histone marks (H3K4me1/2) in neural progenitor cells (NPCs) or endomesoderm cells. RNA polymerase II (PolII) chromatin interaction analysis with paired-end tagging (ChIA-PET) [15] showed that the target genes of these regulatory regions were indeed significantly upregulated in NPCs. Enhancer/luciferase reporter assays demonstrated that these regions function as in gene activation when 5hmC is removed for these sites. Together, our findings suggest that 5hmC is as a novel marker for transcriptional silent enhancers in mESCs for regulatory regions that are activated during development.

## Results

### A group of 5hmC-enriched distal TFBSs is lacking activating histone marks and nascent RNA transcription

A recent survey had found 5hmC enriched at TFBSs in hESC [11], mouse neuronal cells, and adipocytes [7]. Therefore, we investigated 5hmC levels [13] at the binding sites of 13 key transcription factors (TFs) (Nanog, Oct4, STAT3, Smad1, Sox2, Zfx, c-Myc, n-Myc, Klf4, Esrrb, Tcfcp2l1, E2f1 and CTCF) in mESC [16]. We confirmed previous results [11, 12] that 5hmC was generally depleted at the core of the proximal (within 2 kb to transcription start sites (TSSs)) TFBSs, but relatively high in the regions neighboring (±2 kb) the core (Additional file 1: Figure S1A). We also confirmed that 5hmC is highly enriched at the core of distal binding sites of many TFs, such as Zfx and Esrrb (Additional file 1: Figure S1B) [11, 12].

**Figure 1:**
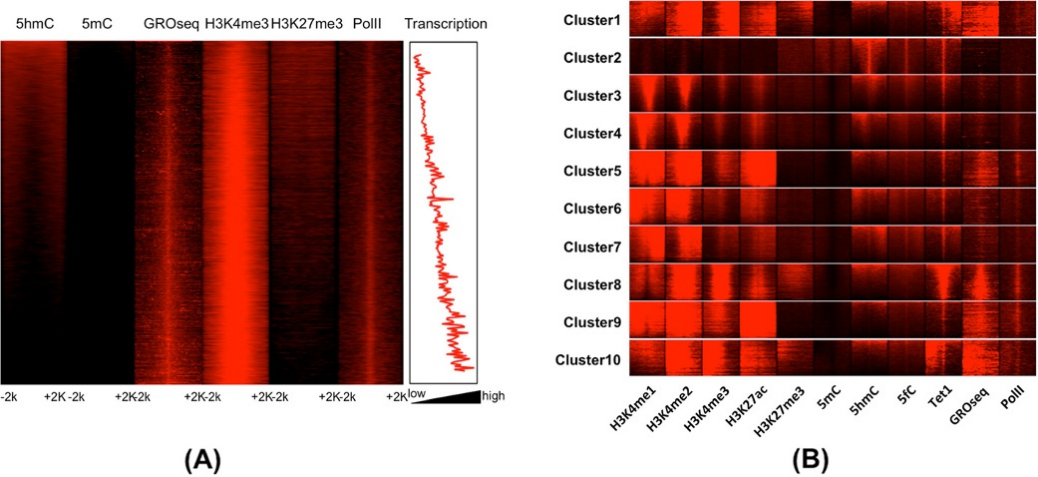
**5hmC and other epigenetic modifications in ESCs. (A)** Correlation between 5hmC and various marks. The TFBSs were sorted based on the 5hmC levels in ±2 K regions relative to the center of the binding sites. 5hmC levels at promoter-proximal TFBSs were positively correlated with H327me3 levels and inversely correlated with GROseq and PolII levels. Transcription levels of the genes associated with the promoter were calculated using GROseq . In the sorted list, we averaged the transcription levels of the adjacent 100 genes. **(B)** Clustering results of 5hmC with other epigenomic data at distal (>2kbp from known TSSs) TFBSs. Cluster 1, 8 and 10 are enriched for H3K4me3 and GROseq, showing the properties of promoters. Cluster 5 and 9 display high levels of H3K27ac, indicative of active enhancers. Cluster 2 is enriched for 5hmC and 5fC, has very low GROseq levels, and lacks all investigated histone marks.

To further investigate the role of 5hmC in gene regulation in conjunction with other epigenetic marks, we performed an integrative analysis using 5hmC, 5mC [13], Tet1 [10], H3K4me1/2/3, H3K27me3, RNA polymerase (Pol) II occupancy [17] and nascent RNAs from global run-on sequencing (GROseq) [18] data. We found that 5hmC levels were inversely correlated with nascent RNA transcription and Pol II occupancy at proximal TFBSs (Figure 1). We confirmed the levels of 5hmC positively correlated with the levels of the repressive H3K27me3 histone mark at proximal TFBSs [8, 12].

To study the epigenetic landscapes surrounding distal TFBSs, we applied the K-means algorithm (K = 10) and found clusters marked by various epigenetic modifications (Figure 1B). Clusters 1, 8 and 10 showed the properties of active promoters: H3K4me2/3 enrichment with relatively low levels of H3K4me1 and the presence of nascent RNA transcripts. These clusters thus likely represent the promoters of long intergenic non-coding RNAs [19] or un-annotated promoters of protein-coding genes. Clusters 5 and 9 showed H3K4me1 and H3K27ac enrichment, indicating active enhancers. These clusters, as well as clusters 3, 4, 6, and 7, showed only a small amount of nascent transcripts or enhancer RNAs (eRNAs), which have been known to correlate with the gene transcription levels of adjacent genes [20, 21]. The presence of eRNAs in these clusters suggest that the TFBS at these clusters have an activating role.

We were especially interested in cluster 2, which was enriched for 5hmC, but was depleted of eRNAs. Strikingly, this cluster had no activating histone marks such as H3K4me1 or H3K27ac [22–24], even though TFs bind at these sites (Figure 1B and Additional file 1: Figure S2). 5mC was depleted at the core of the TFBS, consistent with the previous observation in hESCs [25]. Compared with other clusters, cluster 2 was characterized by low levels of eRNAs and low PolII occupancy. To confirm the enrichment for 5hmC, we investigated the profile of sequencing data from other independent studies [1, 12–14, 26, 27]. Cluster 2 was enriched for 5hmC consistently for all four independently measured datasets (Additional file 1: Figure S3). We also examined TAB-seq, which provides base-resolution sequencing of 5hmC in mESC [3]. The TAB-seq profile also confirmed enrichment for 5hmC at the core of TFBSs for cluster 2 regions for both strands (Additional file 1: Figure S4). Together, these data suggest that 5hmC combined with absence of H3K4me1 at distal TFBSs marks inactive enhancers.

Surprisingly, cluster 2 is also highly enriched for 5-formylcytosine (5fC) compared with other clusters (Figure 1B). Both 5fC and 5hmC are involved in the active demethylation pathway [28, 29]. Previous genome-wide study using 5fC revealed that 5fC is enriched at enhancers, especially at poised enhancers marked by H3K4me1 without H3K27ac [30]. However, the properties of the cluster 2 regions are novel, as they lack the H3K4me1 mark. This strongly suggests that 5hmC as well as 5fC mark a novel type of “poised” or silenced enhancer at distal regulatory regions where active histone modification marks are absent.

Next, we interrogated the state of the 5hmC mark in other cell types. In hESCs, we also identified a cluster enriched for 5hmC [3] but depleted for both H3K4me1 and H3K27ac at distal DNaseI hypersensitive sites (DHSs) [31] (Additional file 1: Figure S5). As in mESCs, GROseq levels in hESCs [32] were significantly weaker in this cluster (p-value = 1.7e-14). In mature adipocytes, we observed 5hmC [7] enriched at over 20% of PPARγ binding sites [33] (Additional file 1: Figure S6). Surprisingly, PolII occupancy [33] was depleted when 5hmC was enriched (Additional file 1: Figure S6). These data indicate that 5hmC can be a repressive mark at distal regulatory regions regardless of cell type or differentiation state.

Additional file 1: Table S1 lists the number of binding sites for each TF in cluster 2 in mESCs. The majority of the cluster 2 regions were bound by CTCF, Tcfcp2l1 or Esrrb. Fewer binding sites for Oct4, Sox2, and Nanog, the master regulators for self-renewal and pluripotency in ESCs, were observed in cluster 2 [34]. This is consistent with the observation that 5hmC is depleted at highly active enhancers in ESCs. We further investigated if ChIP intensity is lower for the TFBSs in cluster 2. We did not find statistical differences, even though the average profiles of the TFBSs in cluster 2 were slightly lower compared with the TFBSs in other clusters (Additional file 1: Figure S7).

### 5hmC-enriched distal TFBSs are associated with developmental genes

Next, we analyzed the correlation between 5hmC levels and transcriptional activity of the genes closest to the TFBSs for each cluster. To calculate gene transcription levels, we calculated the reads per kilobase per million mapped reads (RPKM) from GROseq (see Methods). The genes mapping to the TFBSs in cluster 2 had strikingly reduced transcription levels compared to the genes in all other clusters (p-value <1.3e-20), even compared to clusters 8 and 10, where the repressive H3K27me3 mark was relatively enriched (Figure 1B).

**Figure 2:**
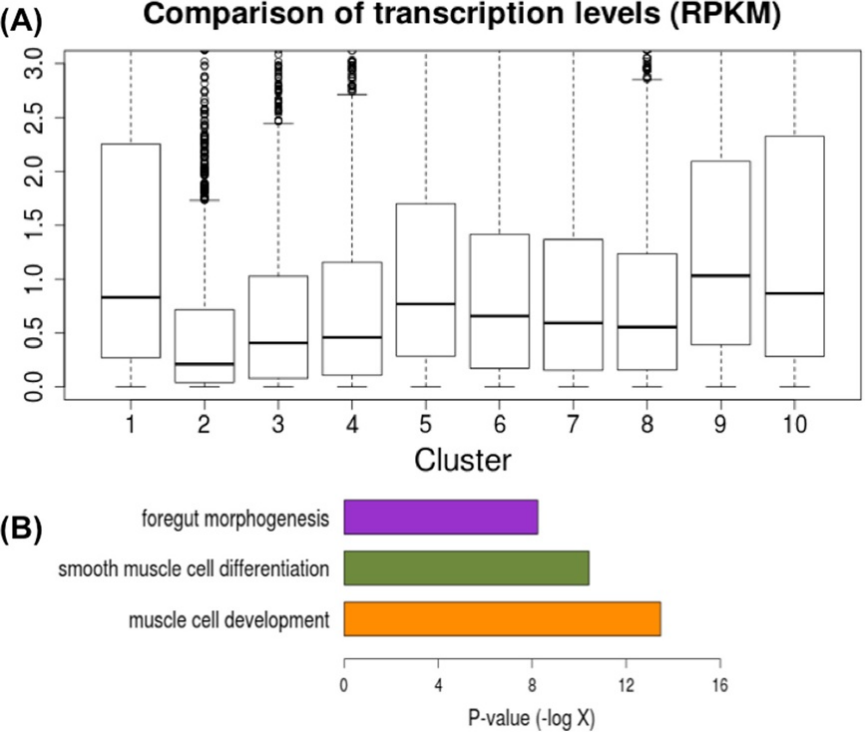
**Functional analysis for TFBSs with 5hmC. (A)** Comparison of the transcription levels of the nearest genes between cluster 2 and the other 9 clusters of distal TFBSs identified in Figure 1B. The transcription levels (RPKM) were calculated using GROseq data. **(B)** GO analysis for the genes close to TFBSs in cluster 2. Organ development terms are enriched.

GO analysis of the genes closest to the TFBSs in cluster 2 using GREAT [35] revealed that the genes in this cluster were enriched for developmental functions, such as “muscle cell development” (p-value = 3.4e-14)” and “foregut morphogenesis” (p-value = 5.8e-9) (Figure 2D). This is consistent with the fact that these genes are silent in ESCs and are only activated once differentiation commences.

**Figure 3:**
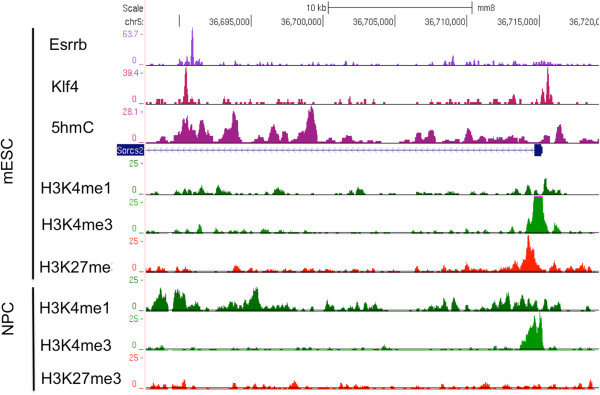
**Chromatin organization at the Sorcs2 gene in mESCs.** The Klf4 and the Esrrb binding sites in the intron of Sorcs2 gene are enriched for 5hmC. These binding sites are depleted with H3K4me1 in mESCs, but are enriched in NPCs.

A snapshot in Figure 3 shows the enrichment for 5hmC at the Klf4 and the Esrrb binding sites located in the first intron of *Sorcs2. Sorcs2* is highly expressed in the developing and mature murine central nervous system [36]. We observed that *Sorcs2* is silent in mESC, and its promoter is bivalently marked by H3K4me3 and H3K27me3 [17]. In mouse neural progenitor cells (NPCs), however, *Sorcs2* is highly expressed [17]. The Klf4 and the Esrrb binding sites are marked by H3K4me1 in NPCs, suggesting an active role of this region as an enhancer during neural development.

### 5hmC-enriched distal TFBSs become activated during development in a lineage-specific way

**Figure 4:**
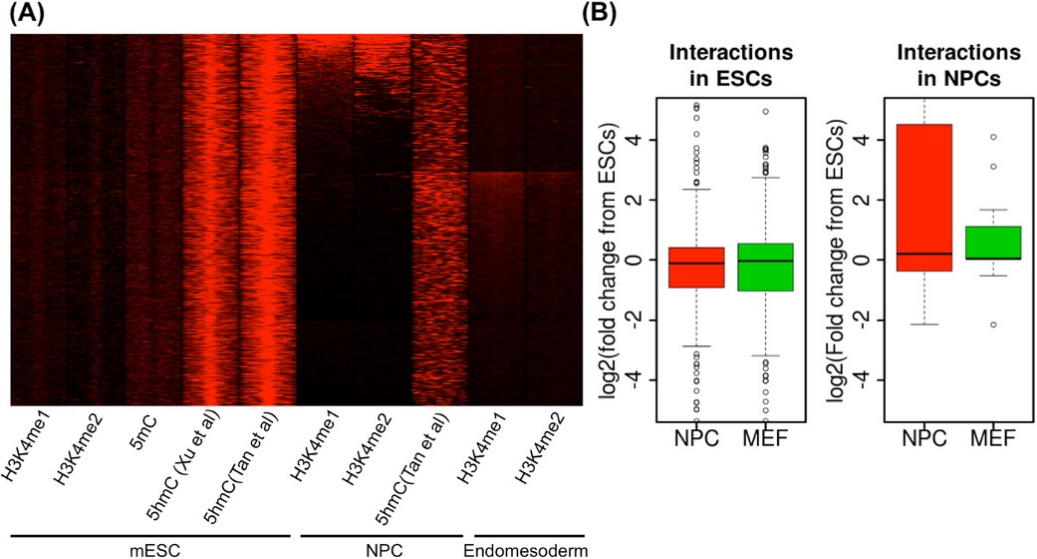
**Lineage specific activation of distal TFBSs in cluster 2. (A)** The enriched H3K4me1/2 in NPCs or endomesoderm cells suggests the potential lineage specific enhancer activation of the TFBSs in cluster 2 after differentiation. **(B)** The target genes of cluster 2 in NPCs identified using ChIA-PET become significantly upregulated (p-value:0.04) when they gained interactions. The expression change in MEF is compared as a control (p-value:0.12).

Because of the coordination of high 5hmC levels with low expression of genes in cluster 2, we hypothesized that 5hmC may attenuate enhancer activity in mESCs, which becomes activated later during development. Therefore, we analyzed H3K4me1/2 data in NPCs [17] and endomesoderm cells [37]. H3K4me1 and H3K4me2 are known to mark enhancers [22]. While H3K4me1/2 enhancer marks were depleted in mESCs, around 9% of distal TFBSs (out of 5,278 TFBSs) showed enriched H3K4me1/2 occupancy in NPCs, and an additional 20% of TFBSs were enriched for H3K4me1/2 in endomesoderm cells (Figure 4A). Overall, 5hmC levels were significantly decreased in cluster 2 regions after differentiation into NPCs [27]. This suggests that a group of 5hmC-enriched enhancers are repressed in mESCs, but selectively become activated during development towards the neuronal or endomesoderm lineage. This implies that other regions in cluster 2 might be activated when ESCs are differentiated into other lineages such as primordial germ cells.

To further determine if the TFBSs in cluster 2 have activating roles in a lineage-specific way, we used chromatin connectivity maps from chromatin interaction analysis with paired-end tagging (ChIA-PET) associated with PolII in ESCs and NPCs [15]. By using the chromatin interaction information, we mapped the target genes of the TFBSs in cluster 2 that were only selectively activated in NPCs. The target genes in ESCs were slightly downregulated in NPC (as well as in mouse embryo fibroblasts (MEFs)) because only a small portion of them become activated in a lineage-specific manner as shown in Figure 4A. In contrast, the target genes in NPCs become significantly upregulated during the transition from ESCs to NPCs (p-value <0.05). Moreover, the changes were significant compared with the target genes for clusters (all p-values were <0.02) (Additional file 1: Figure S9). This further supports the notion that TFBSs in cluster 2 become activated in a lineage specific way following embryonic stem cell differentiation.

### Cluster 2 regions show enhancer activity in mESCs when devoid of 5hmC

**Figure 5:**
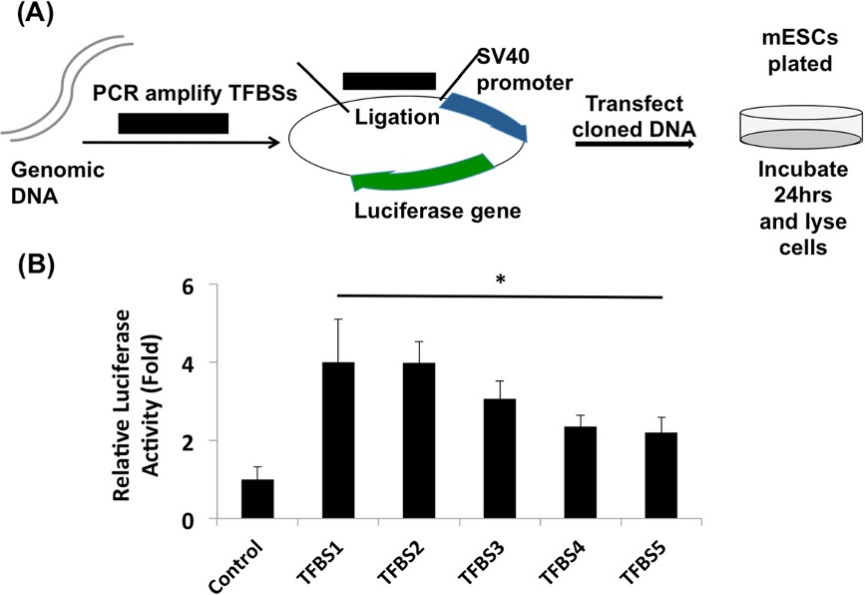
**Enhancer activity of distal TFBSs lacking 5hmC in mESCs. (A)** Schematic diagram of the experimental setup. **(B)** Luciferase reporter assay for 5hmC-enriched putative enhancer regions (about 600 bp) including distal TFBSs in mESCs. Control: empty vector, TFBS1: 5hmC-enriched Klf4/Esrrb binding site (Figure 3), TFBS2: 5hmC-enriched Esrrb/Tcfcp2I1 binding site, TFBS3: 5hmC-enriched Tcfcp2I1 binding site, TFBS4: 5hmC-enriched E2f1 binding site, TFBS5: 5hmC-enriched Nanog/Sox2. The normalized luciferase activity of control is set as 1. *p-value < 0.05.

Next, we directly determined if lack of 5hmC activates enhancer activity of the distal TFBSs in mESCs. We selected five highly 5-hydroxymethylated distal TFBSs regions from cluster 2, including the site in *Sorcs2* (TFBS1). This site is also enriched for ChIA-PET [15] reads. We amplified these sequences (±600 bp) by PCR and subcloned them into luciferase reporter plasmids containing a minimal promoter. We found that these DNA sequences indeed possess enhancer activity in mESCs when lacking 5hmC, showing on average 3-fold increased luciferase activity compared to control (Figure 5). This *in vitro* study suggests that 5hmC-enriched distal TFBSs are bona fide enhancers, which are silent in mESCs when marked with 5hmC.

We also investigated if 5hmC at distal regulatory regions has a repressive role using the Tet1 shRNA suppression experiments in mESCs [38]. We found that the target genes of cluster 2 were significantly upregulated (p-value < 0.01) after Tet1 gene suppression, suggestive of repressive roles of 5hmC (Additional file 1: Figure S9). We did not find a similar pattern in Tet2 shRNA-treated mESCs, possibly due to the fact that Tet2 is dominantly associated with the 5hmC present in gene bodies [38].

## Discussion

The field of DNA methylation has expanded recently, with the identification of multiple cytosine variants; 5hmC, 5fC, and 5-carboxylcytosine (5-caC) [28, 39]. Among the cytosine variants, 5hmC has been most extensively studies [1, 3, 9–14]. Although there are genome-wide 5hmC maps in several cell types, our understanding about the functional role of 5hmC remains limited.

The contribution of the 5hmC modification to gene regulation is actively debated. Recent studies found that 5hmC gain is accompanied by H3K27me3 loss at promoters and in the gene body during neurogenesis, suggesting an activating role of 5hmC [40]. On the other hand, the presence of 5hmC at the promoter of bivalently marked genes [8, 12, 14] and *in vitro* transcription studies revealed a repressive role of 5hmC at promoter regions [41]. However, the role of 5hmC at enhancers has not been investigated thus far.

We observed that enrichment of 5hmC corresponds with the depletion of eRNAs at distal TFBSs. Considering that eRNAs correlate with gene transcription [20, 21], we suggest that low levels of 5hmC at enhancers are required for gene expression. Importantly, we found that a subset of distal TFBSs that carry the 5hmC mark in embryonic stem cells become enriched for the activating histone mark (H3K4me1/2) following differentiation into neural progenitors or endomesoderm, suggesting that distal TFBSs with 5hmC are repressed in mESC but become active enhancers in a lineage-specific manner. Indeed, using ChIA-PET interaction information [15], we found that those regions that gained connections to their target genes were significantly upregulated during differentiation compared with the target genes in other clusters. This suggests that their target genes were repressed in ESCs and become selectively activated in a lineage-specific way.

To ascertain if the proposed “silent enhancers” identified above can indeed function as enhancers we employed luciferase reporter assays. We demonstrated that the novel distal elements, characterized by TF binding, high levels of 5hmC, and absence of the H3K4me1 “enhancer” mark, can indeed function as enhancers in mESCs if they are devoid of the 5hmC modification. This experiment is consistent with the notion that 5hmC could inhibit enhancer activity at a subset of distal TFBSs in mESCs.

Our findings are different from the work of Sérandour and colleagues [7], who had suggested an activating role for 5hmC at distal regulatory regions. They identified 5hmC peaks after differentiation which were surrounded by the activating H3K4me2 mark. However, more than 50% the 5hmC peaks they identified were located at genic regions, where they are known to be associated with gene activation [10, 12, 38, 40, 42]. It is also possible that the 5hmC peaks at distal regions are associated with non-coding RNAs such as long non-coding RNAs (lincRNAs) [43]. Sérandour and colleagues also identified 5hmC at distal PPARγ binding sites [33]. Even though Sérandour and colleagues proposed an activating role of 5hmC at these master regulator in adipocytes, only a portion of PPARγ binding sites were enriched for 5hmC [7]. We revisited their data and found that 5hmC was only present at sited lacking PolII occupancy (Additional file 1: Figure S6), indicating that 5hmC at PPARγ binding sites bears repressive roles in mature adipocytes.

In hESCs, we also identified a group of distal DHSs with strong 5hmC but weak H3K4me1 and H3K27ac (Additional file 1: Figure S5). The GROseq levels were significantly weak for the group with 5hmC (Additional file 1: Figure S5). These lines of evidences suggest a general repressive role of 5hmC at distal regulatory regions.

In ESCs, poised enhancers have been suggested to exist at sites where both activating marks (H3K4me1) and repressive marks (H3K27me3) are enriched, but H3K27ac is depleted [23, 24]. 5fC is enriched in this type of poised enhancers (H3K4me1[+] and H3K27ac[−]) [30]. In contrast to these poised enhancers, we identify a novel group of enhancers with no activating histone marks (H3K4me1[−] and H3K27ac[−]) but enrichment only for 5hmC. Furthermore, this group is strongly enriched for 5fC, even though cluster 2 lacks the H3K4me1 mark (Figure 1). Our results strongly suggest that 5hmC and 5fC can be epigenetic mark for poised or silent enhancers. As shown in our results, many of these enhancers display activating histone marks only after differentiation has occurred (Figure 4). The existence of 5hmC and 5fC also show the active oxidation dynamics at these sites.

We found that 5hmC was enriched at distal PPARγ binding sites in fully differentiated adipocytes. These findings suggest 5hmC as a new marker for poised enhancers even in absence of H3K4me1 and H3K27me3. Additionally, we also found enriched 5hmC in NPC at the subset of the active TFBSs (except for cluster 2) in mESCs (Additional file 1: Figure S10). This may suggest that active enhancers in mESCs are repressed by 5hmC in NPC to remove the enhancer activities in mESCs.

The majority of cluster 2 regions are CTCF binding sites (Additional file 1: Table S2). In general, 5hmC levels negatively correlated with CTCF occupancy in cluster 2 (Additional file 1: Figure S11). After differentiation into NPCs, 5hmC became depleted at these sites even though the binding CTCF remained. At these sites, we did not observe activating H3K4me1 and H3K4me2 marks. However, it is difficult to discuss the role of 5hmCs at these sites, because CTCF takes part in various regulatory roles including transcriptional activation, repression, as well as the formation of higher order chromatin structure [44]. The function of 5hmC in mESCs at CTCF binding sites warrants further study.

## Conclusions

We report a new repressive role for 5hmC in gene regulatory regions in mESCs. The TFBSs enriched for 5hmCs were depleted for nascent transcripts and activating histone modification marks in human and mouse ESCs. Furthermore, the 5hmC levels were inversely correlated with PolII occupancy in mESCs as well as in fully differentiated adipocytes. Our findings indicate that 5hmC has a repressive role at specific distal regulatory regions and suggest that 5hmC is a new epigenetic mark for silenced enhancers.

## Methods

### Experimental crocedures

We used genome-wide GROseq maps [18] and ChIP-seq data for chromatin status [17, 45], PolII occupancy [17], 5mC [10], and Tet1 occupancy [10] in mESCs for our integrated analysis. We employed H3K4me1/2 data from NPC [17] and endomesoderm cells [37] to analyze the fate of our novel 5hmC regions after differentiation. We also included 5hmC from various independent studies [1, 12–14, 26, 27] for our analysis. Additional file 1: Table S1 summarizes all genome-wide datasets we used in our study.

All ChIP-seq data were normalized to 10 reads per kilobase per million mapped reads (RPKM) [46]. For clustering analysis we used Mev V4.8 [47] and applied the K-means clustering algorithm using the Pearson correlation with absolute distance as a metric. To cluster distal TFBs in mESCs, we used the H3K4me1/2/3, H3K27ac, H3K27me and 5hmC levels and generated applied clustering (K = 10). We showed other epigenetic marks and GROseq and PolII next to the identified clusters.

To study the functional roles of 5hmC in various regulatory regions, we employed binding site data of 13 TFs (Nanog, Oct4, STAT3, Smad1, Sox2, Zfx, c-Myc, n-Myc, Klf4, Esrrb, Tcfcp2l1, E2f1 and CTCF) in mESC [16].

To investigate 5hmC and nascent RNA levels across genes, we divided the genes into promoter (from -1Kbp to 500 bp around the annotated start site), 3′ end (from −500 bp to 500 bp around the annotated termination site), and gene body regions (500 bp from the annotated start site to −500 bp from the annotated termination site). For transcription levels, we calculated RPKM using GROseq reads from 500 bp of the annotated start site to the annotated termination site in order not to include transcriptional pausing at promoters [20, 48].

### Luciferase reporter assay

Genomic DNA was prepared from R1 mouse embryonic stem cells [49]. About 600 bp genomic fragments for five distal TFBSs in cluster 2 were amplified by PCR with dNTPs and the PCR products ligated into the pGL3-SV40 luciferase vector (Promega). Empty vector (control) or cloned vectors were transfected directly into R1 mESC, together with the pRL-tk vector (Promega) as internal control, using Lipofectamine LTX (Life Technologies). At 24 h after transfection, cells were harvested and lysates subjected to the dual-luciferase reporter assay (Promega). Firefly luciferase activity was measured and normalized to the internal control, Renilla luciferase activity.

## Electronic supplementary material

Additional file 1: Figure S1: 5hmC profile at promoters and enhancers. **Figure S2.** Comparison of the characteristics of each cluster. **Figure S3.** Comparison of the 5hmC patterns for each cluster. **Figure S4.** The 5hmC profile of cluster 2 using TAB-Seq. **Figure S5.** The 5hmC clusters in hESCs. **Figure S6.** The 5hmC clusters in mature adipocytes [10]. **Figure S7.2** The average profiles of TFs at cluster 2. **Figure S8.** The gene expression change for the target genes for each cluster. **Figure S9.** The gene expression changes of the target genes after Tet1 knockdown for each cluster. **Figure S10.** The 5hmC in mESC and NPC at the TFBSs in mESCs. **Figure S11.** 5hmC at CTCF binding sites in cluster 2. **Table S1.** Datasets. **Table S2.** The frequency of transcription factor occupancy in cluster 2. (DOCX 2 MB)
