# Supplementary material for: 5-hydroxymethylcytosine represses the activity of enhancers in embryonic stem cells: a new epigenetic signature for gene regulation
Source: BMC Genomics. 2014 Aug 9;15(1):670. doi: 10.1186/1471-2164-15-670 (PMC4133056; doi:10.1186/1471-2164-15-670)
Supplement: Supplementary file 1 — Additional file 1: Figure S1: 5hmC profile at promoters and enhancers. Figure S2. Comparison of the characteristics of each cluster. Figure S3. Comparison of the 5hmC patterns for each cluster. Figure S4. The 5hmC profile of cluster 2 using TAB-Seq. Figure S5. The 5hmC clusters in hESCs. Figure S6. The 5hmC clusters in mature adipocytes [10]. Figure S7.2 The average profiles of TFs at cluster 2. Figure S8. The gene expression change for the target genes for each cluster. Figure S9. The gene expression changes of the target genes after Tet1 knockdown for each cluster. Figure S10. The 5hmC in mESC and NPC at the TFBSs in mESCs. Figure S11. 5hmC at CTCF binding sites in cluster 2. Table S1. Datasets. Table S2. The frequency of transcription factor occupancy in cluster 2. (DOCX 2 MB) [file 12864_2014_6350_MOESM1_ESM.docx]

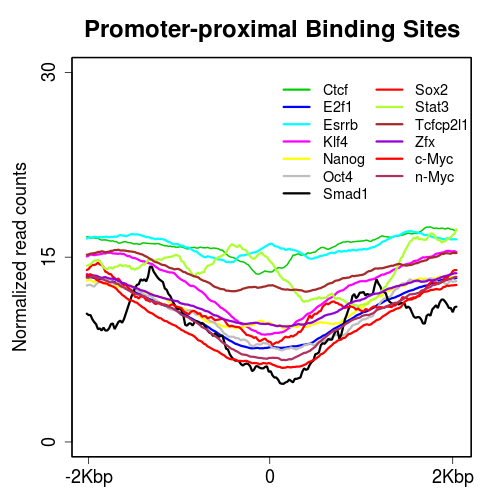


**(A)**


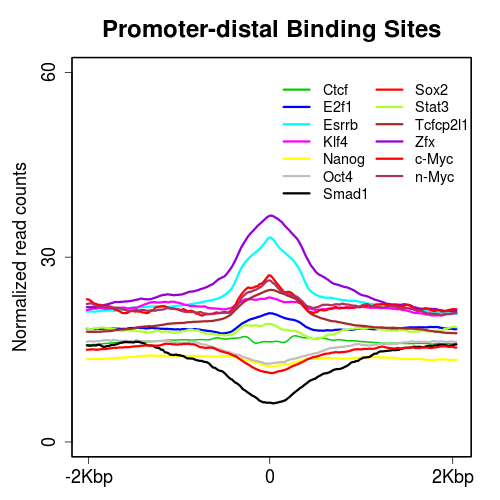


**(B)**

**Figure S1. 5hmC profile at promoters and enhancers.** (A) The average profiles of 5hmC at promoter-proximal TFBSs. 0 represent 2he center of the TFBSs. (B) The average profile of 5hmC at 13 TFBSs in promoter-distal (>2kbps) regions.


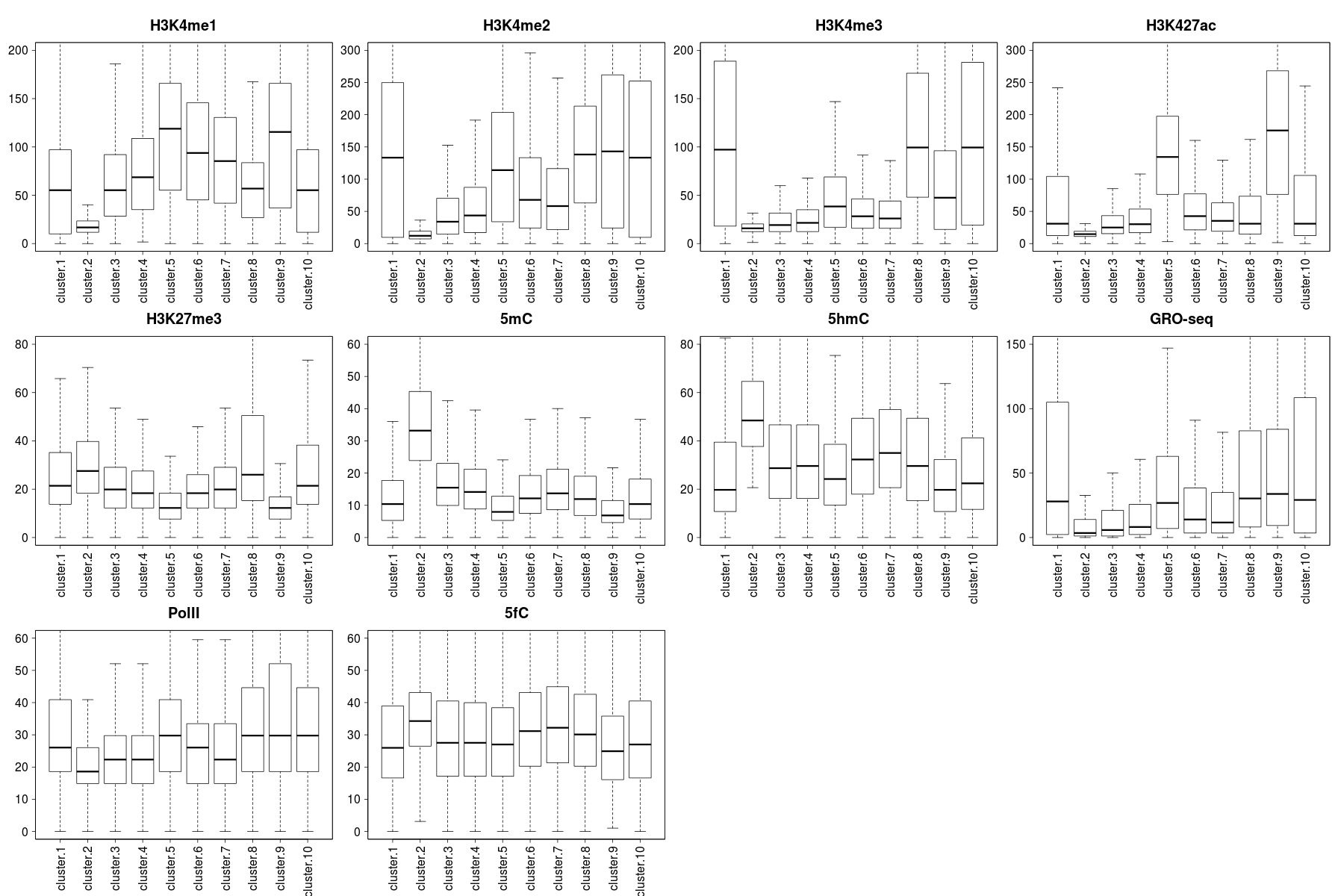


**Figure S2. Comparison of the characteristics of each cluster**. We plotted the levels of H3K4me1/2/3, H3K27ac, H3K27me3, 5mC, 5hmC, GROseq, PolII and 5fC for all clusters. Cluster2 is significantly enriched with 5hmC and 5fC, but depleted for enhancer marks (H3K4me1/2, H3K27ac), eRNA (GROseq) and PolII. The high level of 5mC is due to the enrichment for 5mC at the regions flanking the TFBSs.


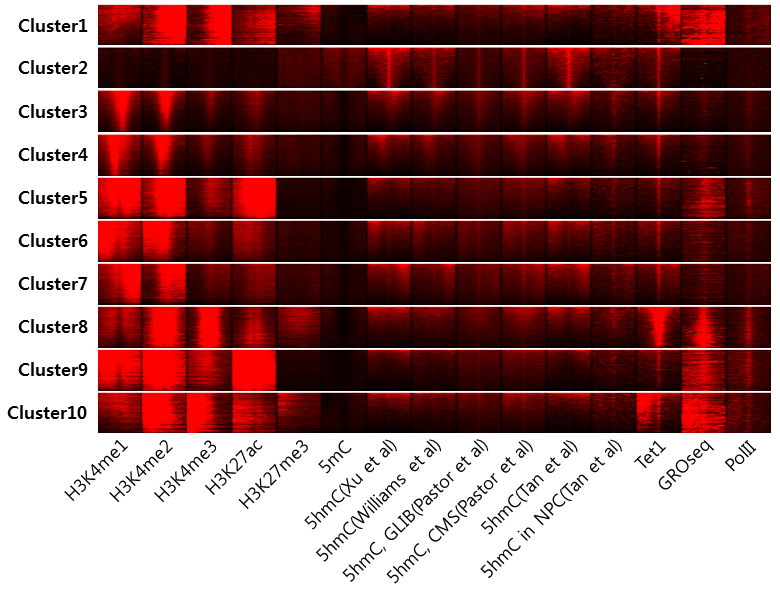


**Figure S3. Comparison of the 5hmC patterns for each cluster.** 5hmC data were obtained from [[1-6](#_ENREF_1)]. Cluster 2 shows consistent enrichment of 5hmC for all four independent studies.


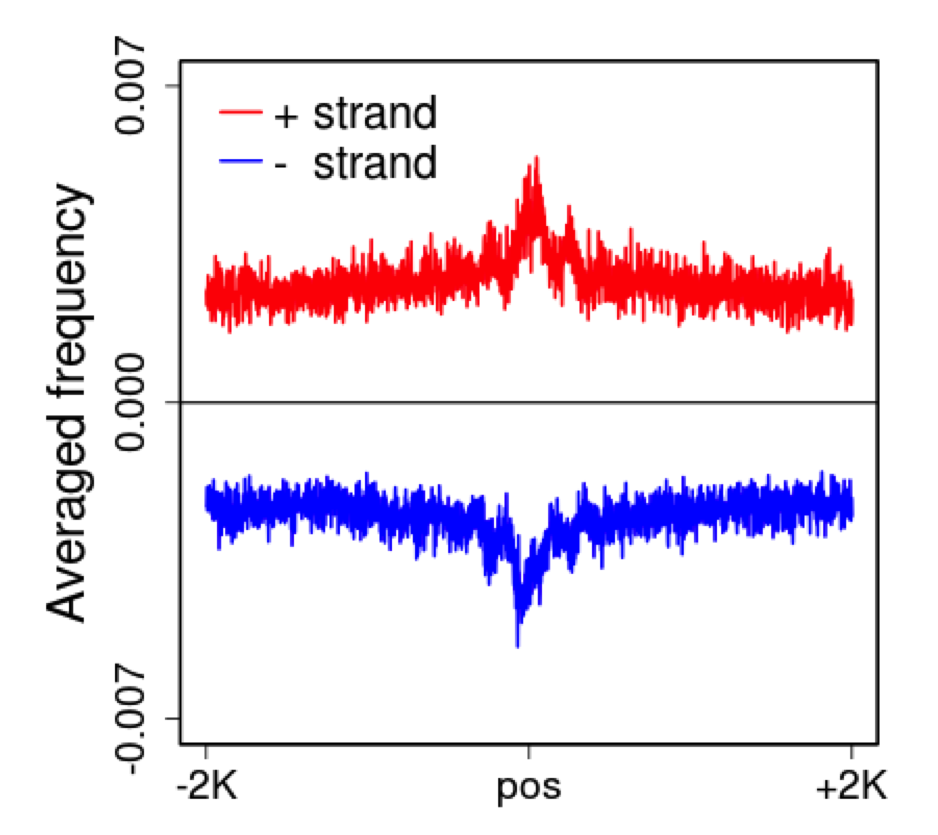


**Figure S4. The 5hmC profile of cluster 2 using TAB-Seq.** The average profiles of 5hmC were generated using TAB-Seq data in mESC. 5hmC is enriched at the center of TFBSs for both strands.


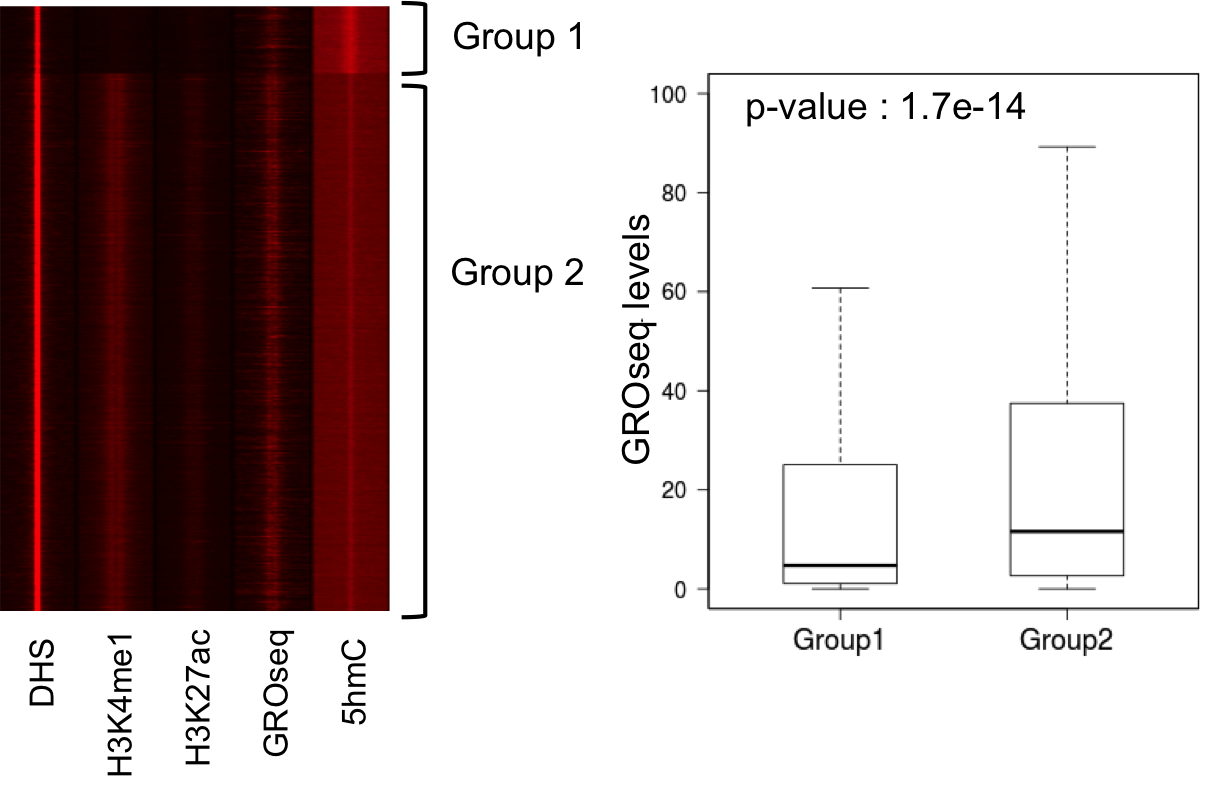


**Figure S5. The 5hmC clusters in hESCs.** We performed clustering using 5hmC [[7](#_ENREF_7)], H3K4me1 and H3K27ac [[8](#_ENREF_8)] at distal DHSs [[8](#_ENREF_8)] in hESCs and identified 2 groups. Among 72,395 distal DHSs, we identified 8,034 sites (Group 1) enriched for 5hmC, while H3K4me1 and H3K27ac were depleted. GROseq levels [[9](#_ENREF_9)] were significantly weak in this cluster.


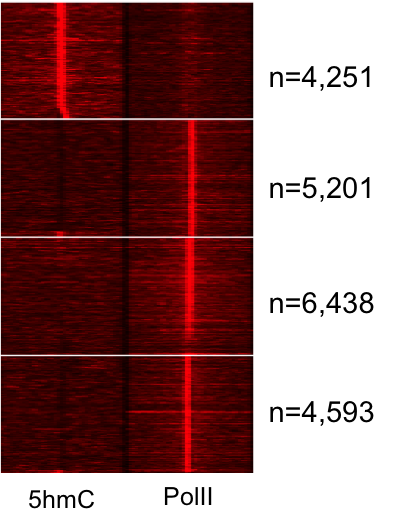


**Figure S6. The 5hmC clusters in mature adipocytes [**[**10**](#_ENREF_10)**].** We clustered 5hmC with PolII [[11](#_ENREF_11)] using the K-means clustering (K=4) algorithm at distal PPARγ binding sites [[11](#_ENREF_11)]. 5hmC and PolII were exclusively observed at these enhancers.


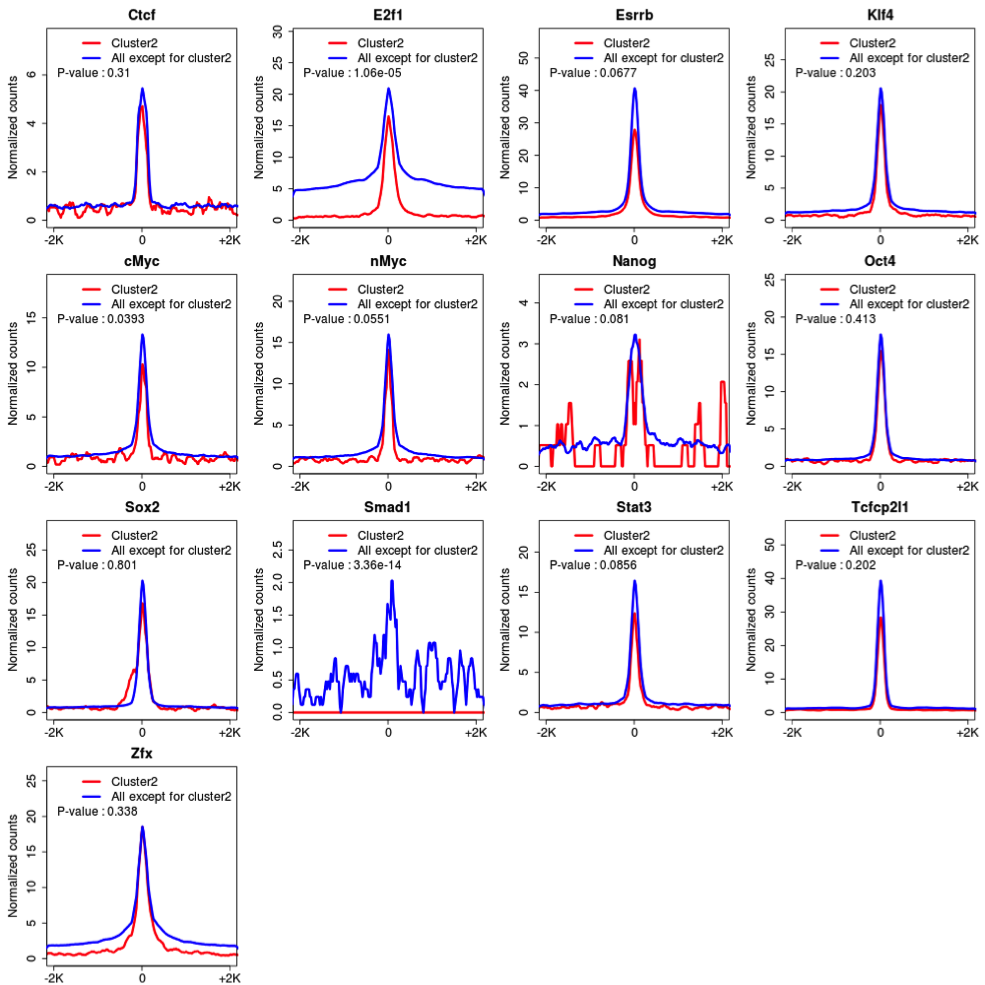


**Figure S7. The average profiles of TFs at cluster 2.** The average profiles of 13 TFs in cluster 2 and other clusters. The p-value from t-test was calculated using the signals around ±100bps. The significant p-values for Smad1 is because of the low number (2) of binding sites and for E2f1 the flanking signals around the binding sites.


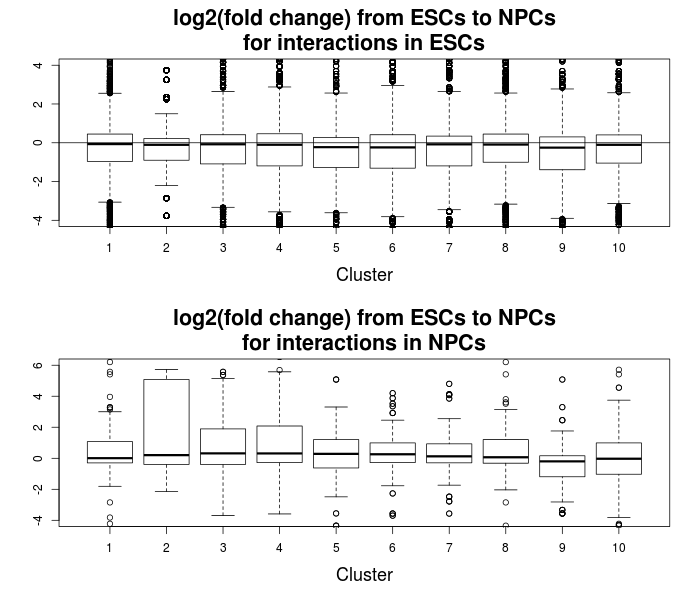


**Figure S8. The gene expression change for the target genes for each cluster.** Using ChIA-PET, we obtained the target genes in ESCs and in NPCs. The genes with interacting chromatin in ESCs overall down-regulated. The target genes of cluster 2 significantly upregulated compared with other clusters (all p-values<0.02). (* if p-value<0.001 and ** if p-value <0.0001)


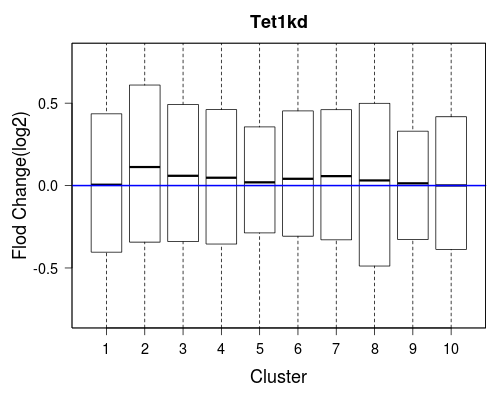

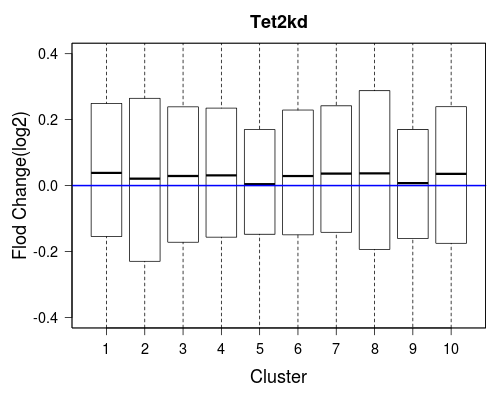


**Figure S9. The gene expression changes of the target genes after Tet1 knockdown for each cluster.** Fold changes were calculated using RNAseq data after Tet1 knockdown in mESCs [[12](#_ENREF_12)]. After knockdown of Tet1, the changes were significant in cluster 2 (p-value: 0.006). For other clusters, p-value>0.01.


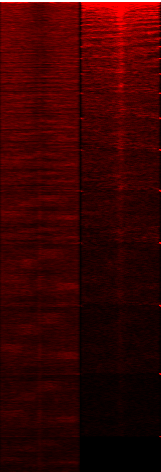


5hmC in mESC

5hmC in NPC

**Figure S10. The 5hmC in mESC and NPC at the TFBSs in mESCs.** TFBS from all clusters (except for cluster 2) were sorted based on the 5hmC levels in NPC. We used 5hmC in mESCs and NPC from [[6](#_ENREF_6)].


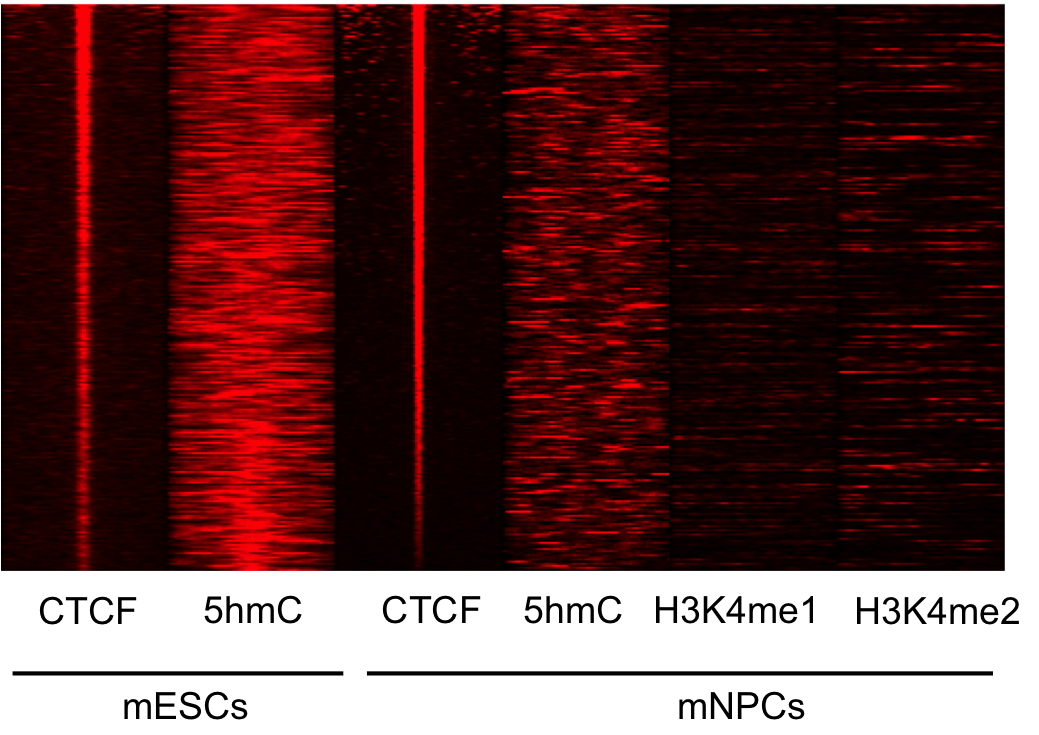


**Figure S11. 5hmC at CTCF binding sites in cluster 2.** 5hmC levels were shown at the CTCF binding sites in cluster 2. After differentiation into NPCs, 5hmC become depleted.

**Table S1. Datasets.**

| Cell type | Data type | GEO accession number | Reference |
| --- | --- | --- | --- |
| mESCs (E14) | ChIP-seq (Nanog, Oct4, STAT3, Smad1, Sox2, Zfx, c-Myc, n-Myc, Klf4, Esrrb, Tcfcp2l1, E2f1 and CTCF) | GSE11431 | [[13](#_ENREF_13)] |
| mESCs (E14) | MeDIP-seq : 5mC  ChIP-seq : H3K27ac | GSE38596  GSE36114 | [[14](#_ENREF_14)]  [[15](#_ENREF_15)] |
| mESC (E14)  NSC, NPC | ChIA-PET | GSE44067 | [[16](#_ENREF_16)] |
| mESC(E14)-Endomesoderm | ChIP-seq : H3K4me1, H3K4me2 | GSE36114  GSE38596 | [[15](#_ENREF_15)] |
| mESCs( E14TG2a.4) | 5hmC (Williams) | GSE24841 | [[5](#_ENREF_5)] |
| mESCs(E14Tg2A) | Bisulfite-seq : 5hmC | GSE36173 | [[7](#_ENREF_7)] |
| mESCs(J1/E14) | TET1,MeDIP-Seq: 5hmC | GSE28500 | [[3](#_ENREF_3)] |
| mESCs (J1) | MeDIP-Seq: 5mC, 5hmC | EBI: EPR000570 | [[3](#_ENREF_3)] |
| mESCs (V6.5) | GROseq | GSE27037 | [[17](#_ENREF_17)] |
| mESCs (V6.5) | ChIP-seq : H3K4me1, H3K4me2, H3K4me3, H3K27me3, PolII | GSE11172  GSE12241 | [[18](#_ENREF_18), [19](#_ENREF_19)] |
| mESCs (V6.5) | 5hmC(Pastor)-GLIB  5hmC(Pastor)-CMS | GSE28682 | [[4](#_ENREF_4)] |
| mESCs(V6.5) | RNA-seq : Tet1kd, Tet2kd | GSE50198 | [[12](#_ENREF_12)] |
| mESCs (*Tdg*^fl/fl^) | 5fC | GSE41545 | [[20](#_ENREF_20)] |
| mESCs(46C) | MeDIP-Seq : 5hmC (Tan) | GSE40810 | [[6](#_ENREF_6)] |
| mNPCs | MeDIP-Seq : 5hmC (Tan) | GSE40810 | [[6](#_ENREF_6)] |
| \|  \|  \| \| --- \| --- \|   Mouse adipocyte(3T3L1) | ChIP-seq : PPAR$\gamma$, PolII | GSE13511 | [[11](#_ENREF_11)] |
| hESCs (H1) | Bisulfite-seq : 5hmC | GSE36173 | [[7](#_ENREF_7)] |
|  | H3K4me1, H3K27ac, DNaseI | ENCODE | [[8](#_ENREF_8)] |
|  | GROseq | GSE41009 | [[9](#_ENREF_9)] |

**Table S2. The frequency of transcription factor occupancy in cluster 2.** Hyper-geometric p-values were calculated.

|  | Number of TFBSs in cluster 2 | Total number of distal TFBSs in mESCs | Ratio | p-value |
| --- | --- | --- | --- | --- |

| CTCF | 2,728 | 27,051 | 10.08% | <1e-130 |
| --- | --- | --- | --- | --- |

| E2f1 | 73 | 9,730 | 0.75% | 1 |
| --- | --- | --- | --- | --- |
| Esrrb | 1,004 | 14,102 | 7.12% | 4e-24 |
| Klf4 | 108 | 5,752 | 1.88% | 1 |
| cMyc | 5 | 861 | 0.58% | 1 |
| nMyc | 24 | 2,158 | 1.11% | 1 |
| Nanog | 128 | 8,912 | 1.44% | 1 |
| Oct4 | 33 | 2,674 | 1.23% | 1 |
| Sox2 | 29 | 3,919 | 0.74% | 1 |
| Smad1 | 2 | 1,032 | 0.19% | 1 |
| STAT3 | 33 | 1,867 | 1.77% | 1 |
| Tcfcp2l1 | 1,026 | 17,881 | 5.73% | 0.0017 |
| Zfx | 85 | 3,881 | 2.19% | 1 |

**References**

1. Wu H, Zhang Y: **Tet1 and 5-hydroxymethylation: a genome-wide view in mouse embryonic stem cells**. *Cell Cycle* 2011, **10**(15):2428-2436.

2. Wu H, D'Alessio AC, Ito S, Wang Z, Cui K, Zhao K, Sun YE, Zhang Y: **Genome-wide analysis of 5-hydroxymethylcytosine distribution reveals its dual function in transcriptional regulation in mouse embryonic stem cells**. *Genes Dev* 2011, **25**(7):679-684.

3. Xu Y, Wu F, Tan L, Kong L, Xiong L, Deng J, Barbera AJ, Zheng L, Zhang H, Huang S *et al*: **Genome-wide regulation of 5hmC, 5mC, and gene expression by Tet1 hydroxylase in mouse embryonic stem cells**. *Mol Cell* 2011, **42**(4):451-464.

4. Pastor WA, Pape UJ, Huang Y, Henderson HR, Lister R, Ko M, McLoughlin EM, Brudno Y, Mahapatra S, Kapranov P *et al*: **Genome-wide mapping of 5-hydroxymethylcytosine in embryonic stem cells**. *Nature* 2011, **473**(7347):394-397.

5. Williams K, Christensen J, Pedersen MT, Johansen JV, Cloos PA, Rappsilber J, Helin K: **TET1 and hydroxymethylcytosine in transcription and DNA methylation fidelity**. *Nature* 2011, **473**(7347):343-348.

6. Tan L, Xiong L, Xu W, Wu F, Huang N, Xu Y, Kong L, Zheng L, Schwartz L, Shi Y *et al*: **Genome-wide comparison of DNA hydroxymethylation in mouse embryonic stem cells and neural progenitor cells by a new comparative hMeDIP-seq method**. *Nucleic Acids Res* 2013, **41**(7):e84.

7. Yu M, Hon GC, Szulwach KE, Song CX, Zhang L, Kim A, Li X, Dai Q, Shen Y, Park B *et al*: **Base-resolution analysis of 5-hydroxymethylcytosine in the Mammalian genome**. *Cell* 2012, **149**(6):1368-1380.

8. Bernstein BE, Stamatoyannopoulos JA, Costello JF, Ren B, Milosavljevic A, Meissner A, Kellis M, Marra MA, Beaudet AL, Ecker JR *et al*: **The NIH Roadmap Epigenomics Mapping Consortium**. *Nat Biotechnol* 2010, **28**(10):1045-1048.

9. Sigova AA, Mullen AC, Molinie B, Gupta S, Orlando DA, Guenther MG, Almada AE, Lin C, Sharp PA, Giallourakis CC *et al*: **Divergent transcription of long noncoding RNA/mRNA gene pairs in embryonic stem cells**. *Proceedings of the National Academy of Sciences of the United States of America* 2013, **110**(8):2876-2881.

10. Serandour AA, Avner S, Oger F, Bizot M, Percevault F, Lucchetti-Miganeh C, Palierne G, Gheeraert C, Barloy-Hubler F, Peron CL *et al*: **Dynamic hydroxymethylation of deoxyribonucleic acid marks differentiation-associated enhancers**. *Nucleic Acids Res* 2012, **40**(17):8255-8265.

11. Nielsen R, Pedersen TA, Hagenbeek D, Moulos P, Siersbaek R, Megens E, Denissov S, Borgesen M, Francoijs KJ, Mandrup S *et al*: **Genome-wide profiling of PPARgamma:RXR and RNA polymerase II occupancy reveals temporal activation of distinct metabolic pathways and changes in RXR dimer composition during adipogenesis**. *Genes Dev* 2008, **22**(21):2953-2967.

12. Huang Y, Chavez L, Chang X, Wang X, Pastor WA, Kang J, Zepeda-Martinez JA, Pape UJ, Jacobsen SE, Peters B *et al*: **Distinct roles of the methylcytosine oxidases Tet1 and Tet2 in mouse embryonic stem cells**. *Proceedings of the National Academy of Sciences of the United States of America* 2014, **111**(4):1361-1366.

13. Chen X, Xu H, Yuan P, Fang F, Huss M, Vega VB, Wong E, Orlov YL, Zhang W, Jiang J *et al*: **Integration of external signaling pathways with the core transcriptional network in embryonic stem cells**. *Cell* 2008, **133**(6):1106-1117.

14. Yu P, Xiao S, Xin X, Song CX, Huang W, McDee D, Tanaka T, Wang T, He C, Zhong S: **Spatiotemporal clustering of the epigenome reveals rules of dynamic gene regulation**. *Genome Res* 2013, **23**(2):352-364.

15. Xiao S, Xie D, Cao X, Yu P, Xing X, Chen CC, Musselman M, Xie M, West FD, Lewin HA *et al*: **Comparative epigenomic annotation of regulatory DNA**. *Cell* 2012, **149**(6):1381-1392.

16. Zhang Y, Wong CH, Birnbaum RY, Li G, Favaro R, Ngan CY, Lim J, Tai E, Poh HM, Wong E *et al*: **Chromatin connectivity maps reveal dynamic promoter-enhancer long-range associations**. *Nature* 2013, **504**(7479):306-310.

17. Min IM, Waterfall JJ, Core LJ, Munroe RJ, Schimenti J, Lis JT: **Regulating RNA polymerase pausing and transcription elongation in embryonic stem cells**. *Genes Dev* 2011, **25**(7):742-754.

18. Mikkelsen TS, Ku M, Jaffe DB, Issac B, Lieberman E, Giannoukos G, Alvarez P, Brockman W, Kim TK, Koche RP *et al*: **Genome-wide maps of chromatin state in pluripotent and lineage-committed cells**. *Nature* 2007, **448**(7153):553-560.

19. Meissner A, Mikkelsen TS, Gu H, Wernig M, Hanna J, Sivachenko A, Zhang X, Bernstein BE, Nusbaum C, Jaffe DB *et al*: **Genome-scale DNA methylation maps of pluripotent and differentiated cells**. *Nature* 2008, **454**(7205):766-770.

20. Song CX, Szulwach KE, Dai Q, Fu Y, Mao SQ, Lin L, Street C, Li Y, Poidevin M, Wu H *et al*: **Genome-wide profiling of 5-formylcytosine reveals its roles in epigenetic priming**. *Cell* 2013, **153**(3):678-691.

.
